# Transdiagnostic Neuroimaging of Depressive and Psychotic Disorders: Applications and Methods

**DOI:** 10.1155/da/9781201

**Published:** 2025-10-28

**Authors:** Drozdstoy Stoyanov, Vince D. Calhoun, Georg Northoff

**Affiliations:** ^1^Medical University Plovdiv, Department of Psychiatry and Medical Psychology, Research Institute & Strategic Research and Innovation Program for the Development of MU-PLOVDIV-(SRIPD-MUP), European Union-NextGenerationEU, Plovdiv 4002, Bulgaria; ^2^Center for Translational Research in Neuroimaging and Data Science 55 Park Place NE, Atlanta GA 30303, USA; ^3^Ottawa Institute of Mental Health Research, 1145 Carling Avenue, Ottawa, Canada

The application of neuroimaging techniques in psychiatry is relevant to the context of the overall progress in neuroscience. It reveals common and specific patterns of structural and functional deterioration of brain networks in mental disorders, which are further correlated with clinical diagnosis. One major challenge remains the incorporation of neuroimaging findings into clinical reasoning. A premise for this caveat is the problematic diagnostic validity of the current systems for classification and diagnosis, which are exclusively based on interviews or self-assessment scales. By definition, depression as part of affective disorders and psychotic disorders are regarded as discrete diagnostic groups in conventional taxonomic systems, whereas from a phenomenological perspective they actually constitute a broader continuum with at least partially shared clinical features and syndromes. The other premise is the controversial body of evidence in neuroscience with high inter and intraindividual variability, which prevents it from adequate integration into clinical diagnosis in psychiatry. The high level of discrepancy between methods and methodological approaches contributes further to the so-called explanatory gap between brain and symptoms.

The aim of this special issue is to bring together contributions that address the described challenges in the field in terms of multimodal and multivariate neuroscience and clinical data integration, including various methods for semi-unsupervised machine learning to produce novel diagnostic classes and therapeutic targets. Critically, we aim at more careful insights into translational research and data management, which can help to converge clinical assessments with neuroimaging or other biological tests to overcome the conventional dichotomy between depression and psychotic disorders.

The study of Markin et al. [https://doi.org/10.1155/da/5974860] demonstrates a possible functional neuroimaging basis for altered temperamental traits in patients with bipolar disorder. They align with previous reports about functional brain connectivity implicated in the stress-diathesis explanatory model of schizophrenia [1]. It is evident in that context that the alterations of functional connectivity at rest and the relevant psycho-biological model of personality as state-independent (trait) measure may underpin the two major diagnostic groups of severe mental disorders.

The findings of Korotokov et al. on functional MRI correlates of state-dependent measures [https://doi.org/10.1155/da/2617054] are both convergent and divergent with existing literature. Convergent findings relate to activations of the precuneus (PRC), superior parietal lobule, and inferior parietal lobule during depression scale item response in patients with depression. This contributes to the conceptualization of depression as a cognitive dysfunction. Moreover, established connections between the PRC, visual cortex, and medial temporal lobe support the hypothesis that this network is crucial for constructing and regulating internal models of reality, processes that are often disrupted in affective disorders [2]. Divergent findings in this study concern the role of the middle frontal gyrus (MFG). However, there is evidence of functional connectivity between PRC and MFG [3], linking two core hubs of the default mode network (DMN) and central executive network, respectively. Given the PRC's role in self-referential processing and the MFG's in cognitive control, a dynamic balance between these regions is essential for optimal neural processing and cognitive function. This balance could be indirectly regulated by the perception of the internal and external world, such that accurate sensorimotor integration by the PRC informs and modulates MFG activity. That means that the observed activations do not necessarily contradict previous results, but are rather complementary, reflecting yet another aspect of the dialectic balance between MFG and PRC.

The results from the next contribution in this special issue [https://doi.org/10.1155/da/2848929] suggest that the dynamic changes in the DMN, dorsolateral prefrontal cortex (DLPFC) connectivity predict the effect of real-time functional MRI neurofeedback in the treatment of auditory hallucinations. This evidence is correlated with earlier progress in the field, explaining the mechanisms of AH [4] and may be used to inform and navigate therapeutic strategy in patients suffering from AH.

Subthreshold affective syndromes, including sub-threshold depression (SD), are of paramount significance in terms of timely identification and proper diagnostic assessment. In the next study from this special issue [https://doi.org/10.1155/da/7645625], functional connectivity and functional near-infrared spectroscopy have been applied for the classification of individuals with SD. Those data provide insights into the underlying neural circuits' aberrations and guidance for objective early diagnosis and prevention of major depression.

The next two contributions of the special issue are relevant to the translational neuroimaging in patients with breast cancer and comorbid emotional dysfunction [https://doi.org/10.1155/2024/9294268].

The authors report that psychological resilience can predict depressive disorder and can mediate the association between brain connectome signatures and depression in patients diagnosed with breast cancer. Furthermore, anisotropy in the inferior fronto–occipital fasciculus (IFOF) is determined as a potential biomarker for demoralization in patients with newly diagnosed breast cancer [https://doi.org/10.1155/2024/5595912].

The findings of Lee and associates [https://doi.org/10.1155/da/9062022] indicate the application of graph neural networks to investigate distinct neural mechanisms underlying major depression and schizophrenia. These results outline the capacity of graph-based models for the introduction of neuroscience-informed revision of psychiatric classifications, alongside other machine learning methods [5, 6].

Finally, Erkan Eyrikaya and İhsan Dağ [https://doi.org/10.1155/da/9943590] integrate advanced computational techniques to guide the development of language-based diagnostic tools in psychopathology.

Overall, the contributions of this special issue are aiming at methodological frameshift, potentially transforming the mental health paradigm.

## References

[1] Zwir I., Arnedo J., Mesa A. (2023). Temperament & Character Account for Brain Functional Connectivity at Rest: A Diathesis-Stress Model of Functional Dysregulation in Psychosis. *Molecular Psychiatry*.

[2] Yamaguchi A., Jitsuishi T. (2024). Structural Connectivity of the Precuneus and Its Relation to Resting-State Networks. *Neuroscience Research*.

[3] Zhang S., Li C.-S. R. (2012). Functional Connectivity Mapping of the Human Precuneus by Resting State fMRI. *Neuroimage*.

[4] Hugdahl K., Craven A. R., Johnsen E. (2023). Neural Activation in the Ventromedial Prefrontal Cortex Precedes Conscious Experience of Being in or Out of a Transient Hallucinatory State. *Schizophrenia Bulletin*.

[5] Pitsik E. N., Maximenko V. A., Kurkin S. A. (2023). The Topology of fMRI-Based Networks Defines the Performance of a Graph Neural Network for the Classification of Patients With Major Depressive Disorder. *Chaos, Solitons & Fractals*.

[6] Stoyanov D., Maes M. H. (2021). How to Construct Neuroscience-Informed Psychiatric Classification? Towards Nomothetic Networks Psychiatry. *World Journal of Psychiatry*.

